# Methadone-induced Damage to White Matter Integrity in Methadone Maintenance Patients: A Longitudinal Self-control DTI Study

**DOI:** 10.1038/srep19662

**Published:** 2016-01-22

**Authors:** Wei Li, Qiang Li, Yarong Wang, Jia Zhu, Jianjun Ye, Xuejiao Yan, Yongbin Li, Jiajie Chen, Jierong Liu, Zhe Li, Wei Wang, Yijun Liu

**Affiliations:** 1 Department of Radiology, Tangdu Hospital, The Fourth Military Medical University, Xi’an, Shaanxi 710038, China; 2 Department of Psychiatry, McKnight Brain Institute, University of Florida College of Medicine, Gainesville, FL 32610, USA

## Abstract

Methadone maintenance treatment (MMT) can induce impairments in brain function and structure, despite its clinical effectiveness. However, the effect of chronic MMT on brain white matter (WM) is not fully known. Thirty-three MMT patients underwent diffusion tensor imaging (DTI) twice – at the start of the study (Scan_1_) and one year later (Scan_2_). Tract-based spatial statistics were used to investigate changes in fractional anisotropy (FA), axial diffusivity (AD) and radial diffusivity (RD) between the two scans. The correlations between DTI indices and methadone consumption and neuropsychological status were analysed. We found significantly decreased FA, decreased AD and increased RD in Scan_2_ in extensive WM regions; overlapping regions were found in the left posterior limb and the retrolenticular part of internal capsule, superior and posterior corona radiata, bilateral external capsule and the right superior longitudinal fasciculus. In addition, the change of FA in the overlapping regions was positively correlated with the accumulated dosage of methadone use, the RD value in Scan_2_ and non-planning impulsiveness (NPI) measured at follow-up. The results suggest that methadone has damaging effects on WM integrity. The dose-dependent pattern and characteristics of the impairment may suggest new strategies for MMT.

Methadone maintenance treatment (MMT) is regarded as the most effective treatment for opioid addiction. Approximate 337,000 patients with heroin abuse have received MMT in China since the initiation of the MMT program in 2004^1^. Long-term MMT benefits heroin addicts by reducing heroin dependence, preventing the spread of HIV and improving the abstinence rates and life status of heroin addicts^2^. However, as a long-acting synthetic opioid drug, despite its therapeutic effectiveness, its potential to induce neuronal adaptations in the brain of methadone users remains uncertain. Meanwhile, emerging evidence suggests that long-term methadone use may lead to adverse consequences for brain structure, metabolism and function and the neuropsychological status of heroin addicted patients.

Toxic effects on neuromechanisms due to long-term opioid consumption have been demonstrated, which indicate that opioids may alter brain structure and function by regulating the genesis of neurons, glia and their precursors in the central nervous system, or by disrupting gliogenesis^3^. Heroin-induced neurotoxic modifications in humans have been demonstrated, while limited observations of methadone-induced neurotoxic effects have been reported. The majority of the previously reported cases were macroscopic lesions observed by neuroimaging, which were induced by acute or subacute use, or an overdose of methadone. The primary magnetic resonance imaging (MRI) characteristics of methadone-induced macroscopic neurotoxic effects have been described as reversible, extensive and symmetric brain hyperintensities in the cerebellum, basal ganglia^4^, deep white matter (WM) of the cerebral hemispheres^5^, subcortical U-fibers^6^, corona radiate and centrum semiovale^7^. Although methadone-related leukoencephalopathy after acute or subacute methadone use has been observed, the influence of chronic methadone exposure remains poorly understood.

Previous research measuring cognitive function has demonstrated several negative effects of long-term MMT. Comparisons of the neuropsychological performance of abstinent heroin addicts and former heroin addicts on MMT have revealed a wide range of impairments in cognitive function in MMT patients, including processing speed, visual-spatial analysis, cognitive flexibility tests, working memory, sustained attention, executive function, verbal and non-verbal learning and resistance to distraction^8^,^9^. However, the underlying neural mechanisms associated with the observed MMT-related cognitive impairments remains uncertain. To the best of our knowledge, only a few studies have focused on the brain’s functional and structural alterations caused by long-term MMT in humans. For instance, one study reported that striatal dopamine transporter uptake was compromised in heroin addicts undergoing MMT compared with that in patients with prolonged abstinence^10^. In a previous study, we observed a decrease in WM integrity induced by methadone in the splenium of the corpus callosum, and a negative correlation between WM integrity and the accumulated dosage of methadone consumed, by applying diffusion tensor imaging (DTI) with the regions of interest (ROI-wise) method^11^. However, the limitations of the previous study make it difficult to interpret the methadone-related negative effects. Specifically, the former study used a group comparison design, in which the effect of previous heroin use could not be eliminated, and did not use quantitative methods to measure WM integrity.

Therefore, the aim of the present study was to explore whether long-term MMT would induce impairments in WM integrity using a quantitative DTI assessment and a longitudinal self-control design. Alterations in WM integrity were assessed by comparing DTI data collected from two scans of each MMT patient over a one year interval. The correlations between WM alterations and methadone use and neuropsychology scores were determined. It was hypothesized that WM integrity in MMT patients would decrease after a one year treatment program, and the observed impairment of WM integrity would be related to the dosage of methadone and neuropsychological scores.

## Results

### Demographic and clinical characteristics

Out of the 50 participants, 12 patients did not complete both of the two scans, and 5 patients’ data were unusable (due to incomplete brain coverage, head motion, or gross cerebral abnormality in T_2_WI structural imaging). Hence, 33 patients who completed both MRI scans were included in the final analysis. The demographic data, heroin addiction history, and MMT status are summarized in Table 1; the depressive, anxious and impulsive scores are summarized in Table 2. All the participants met the initial inclusion criteria of having a stable MMT status; however, all of them were outpatients and reported occasionally using heroin during the interval between the two scans. Therefore, the frequency and accumulated dose of occasional heroin use (which were confirmed by interview and monthly urine tests) were recorded (Table 1).

### Tract-Based Spatial Statistics (TBSS) results

**Figure 1:**
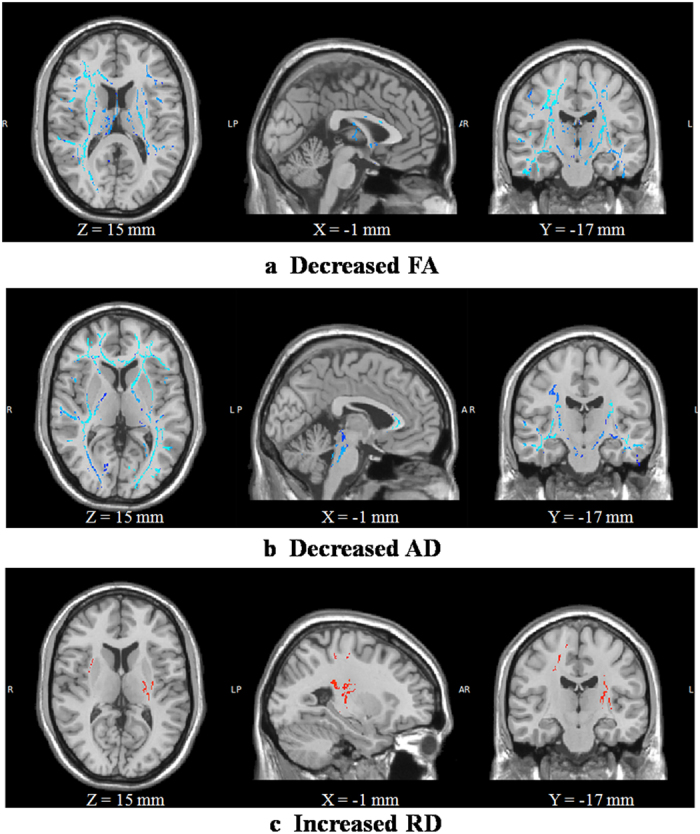
TBSS analysis of FA, AD and RD between S_2_ and S_1_. Compared with S_1_, S_2_ showed significantly decreased FA (**a**), decreased AD (**b**) and increased RD (**c**) in extensive white matter regions (*P* < 0.05, corrected by TFCE and FWE). S_1_ and S_2_ are Scan_1_ and Scan_2_, respectively.

**Figure 2:**
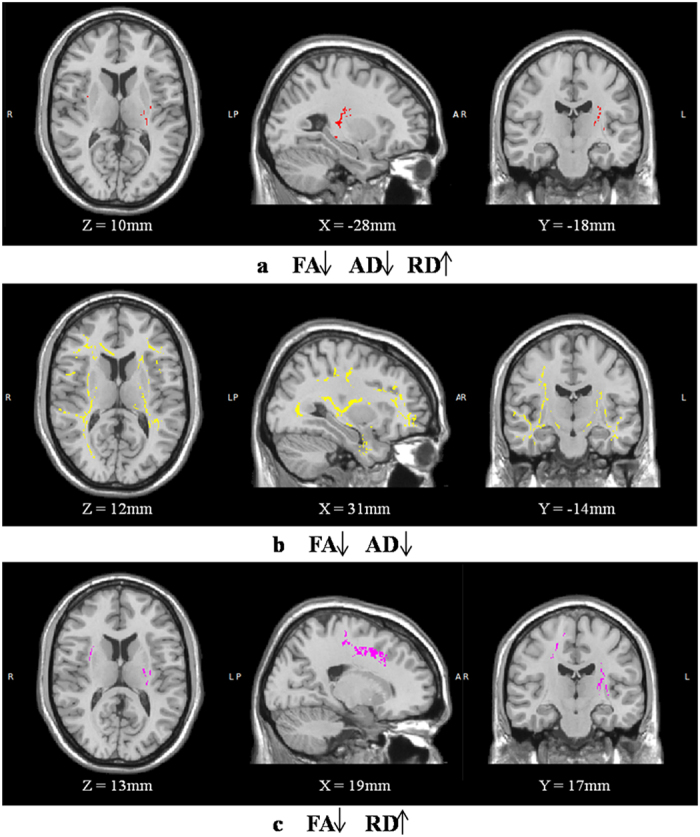
Overlapping white matter regions with changes in FA, AD and RD. White matter regions with decreased FA, decreased AD and increased RD are shown in red (**a**). Regions with decreased FA and decreased AD are shown in yellow (**b**). Regions with decreased FA and increased RD are shown in purple (**c**).

Compared with Scan_1_, Scan_2_ showed significantly decreased FA, decreased AD and increased RD in extensive WM regions (Fig. 1). The overlapping regions with decreased FA, decreased AD and increased RD were mainly located in the left posterior limb and retrolenticular part of the internal capsule, the superior and posterior corona radiata, the bilateral external capsule and the right superior longitudinal fasciculus (Fig. 2a). Regions with decreased FA and decreased AD were mainly located in the bilateral external and internal capsule, superior longitudinal fasciculus, corticospinal tract, right posterior thalamic radiation, anterior corona radiata and the corpus callosum (Fig. 2b). Decreased FA and increased RD were located in the bilateral superior corona radiata, external capsule, corticospinal tract, superior longitudinal fasciculus, left posterior limb and the retrolenticular part of internal capsule (Fig. 2c).

### Correlation results

**Figure 3:**
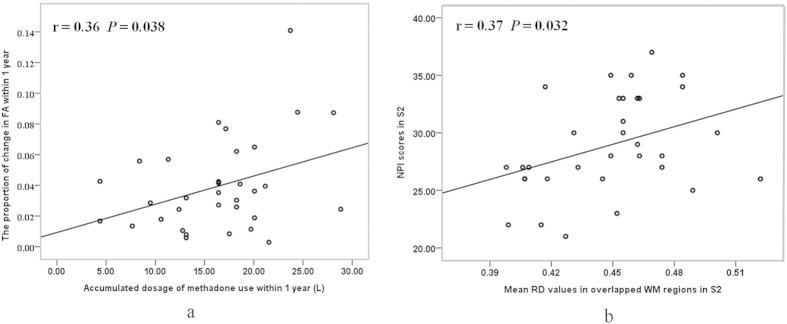
Correlations between DTI indices and clinical characteristics. The proportion of change in FA in the overlapping regions was positively correlated with the accumulated dosage of methadone use within one year (**a**). The RD value in the overlapping regions in S_2_ were positively correlated with the NPI score measured at follow-up (**b**). RD values × 10^−3^ mm^2^/s. S_2_ is Scan_2_.

Correlation analysis indicated that the proportion of change in mean FA values in the overlapping regions with decreased FA, decreased AD and increased RD were positively correlated with the accumulated dose of methadone used (r = 0.36, *P* = 0.038) within one year (Fig. 3a). There were no significant correlations between the changes in the DTI indices and occasional heroin abuse within one year. Although the changes in the DTI indices were not significantly correlated with changes in the neuropsychological scores, we found that the mean RD values in the overlapping regions in Scan_2_ were positively correlated with NPI scores measured at the one year follow-up (r = 0.37, *P* = 0.032) (Fig. 3b). There had been no significant correlation between the DTI indices in Scan_1_ and any of the initial neuropsychological scores.

## Discussion

To the best of our knowledge, this is the first study to provide direct evidence that chronic methadone consumption has damaging effects on WM integrity, using a longitudinal self-control design and quantitative DTI methodology. The results of this study indicate that methadone-treated patients have decreased FA, decreased AD and increased RD in extensive WM regions. The findings are consistent with the hypothesis that chronic use of methadone impairs WM integrity.

Methadone is a highly lipid soluble synthetic opioid, and it is well-absorbed in the brain with a strong affinity for *u*-opioid receptors^12^, which are predominately located in the limbic system and the cerebellum in humans^13^,^14^. Although methadone has similar chemical properties and receptor-mediated actions to heroin, relatively specific regional effects of methadone have been reported. Previously published studies have reported that methadone is distributed mainly in the frontal cortex, hippocampus, cerebellum, and the basal ganglia, as detected by immunohistochemistry in patients with a lethal methadone intake^15^,^16^. Additionally, methadone can produce desensitization in the striatum/accumbens, subiculum, amygdala and periaqueductal gray matter in the rat brain^17^. The WM regions with impaired integrity in the present study were mainly those that form connections between the frontal, temporal, parietal and occipital lobes and the limbic system. The distributions of WM impairment may reveal specific regions where methadone was involved and may help to explain the MMT-induced functional impairment.

DTI analysis is capable of measuring WM integrity, and FA is an important index that is highly sensitive to WM microstructural changes, but not very specific to the characteristics of these changes^18^. To better characterize WM microstructure, multiple diffusion tensor indices (such as AD and RD) should be used^19^. Therefore, the overlapping WM regions with decreased FA, decreased AD and increased RD in the present study may provide more detailed pathological characteristics of WM microstructural changes. It was demonstrated that changes in AD are associated with axon changes, while RD implicates the nature of the myelin^20^. In the human central nervous system, myelin is constructed by oligodendrocytes, which express *u*-opioid receptors^21^. In the present study, significantly increased RD values that hinted of specific demyelination were observed in Scan_2_ compared to Scan_1_, which validated the harmful-effects of methadone on myelin sheets^20^,^22^. The direct effects of methadone on WM integrity remain uncertain, as the changes might have resulted from the demyelination induced by direct impairment or by the activation of an immunological response to neuronal tissues^5^. The results of animal studies indicate that cell death through apoptosis is related to opioid exposure that is induced by mitochondrial dysfunction, oxidative stress, upregulation of the pro-apoptotic proteins Fas, FasL and Bad and a significant loss of mitochondrial membrane potential^23^,^24^,^25^. Opioid-induced apoptosis of microglia cells and neurons in humans has also been reported^26^. Therefore, apoptosis caused by opioid use (including methadone) may induce demyelination and axon impairment.

Several reported impairment mechanisms of methadone, such as respiratory depression and systemic hypoxia, have an indirect influence on WM, which can induce chronic hypoperfusion or hypoxia in the brain. According to the animal model of chronic cerebral hypoperfusion, chronic hypoperfusion can significantly reduce the myelin basic protein, which is regarded as a marker of myelin, and sequentially reduce the neurofilament H, which is considered to be a marker of axonal proteins^27^. Thus, chronic hypoxia may contribute to the pathological characteristics of methadone-related WM impairment, which is currently seen as demyelination accompanied by impairment of the axon.

Impulsiveness has been reported to be associated with substance abuse and related problems, such as relapse^28^,^29^,^30^. NPI has been used to measure the subtypes of impulsiveness, which reflect lack of self-control and intolerance of cognitive complexity^31^. It has been demonstrated that NPI scores are associated with the severity of craving and indirectly related to the possibility of relapse via craving^32^. A positive correlation between RD values in the left posterior limb and the retrolenticular part of internal capsule, superior and posterior corona radiata, bilateral external capsule and the right superior longitudinal fasciculus in Scan_2_ and NPI scores measured at the one year follow-up in this study, may reflect a potential relationship between methadone-related demyelination and increased impulsiveness. However, it is still unclear whether demyelination has a direct effect on the higher heroin relapse rate of MMT patients. Previous studies have observed that methadone exposed infants and children of female heroin users exhibit an altered maturation of WM, which may underlie some of the increased risk for cognitive and behavioral difficulties in these children^33^,^34^. Therefore, it is hypothesized that the WM integrity impairment induced by methadone may be related to the pathological basis of the neuropsychological or functional abnormalities in MMT patients.

In the current study, the FA values in the overlapping regions were positively correlated with the accumulated dosage of methadone use within one year, which may suggest a dose-dependent pattern of the methadone-related WM impairment. Another interesting finding was the relative asymmetry of the FA decrease between the hemispheres; we found that most of the regions with significant FA decreases were located in the right cerebral hemisphere. Previous findings have demonstrated that opioid dependent individuals exhibit a left-greater-than-right asymmetry of cerebral blood flow, which is the opposite of the asymmetry observed in healthy controls^35^. However, whether this left-greater-than-right asymmetry of cerebral blood flow in MMT patients has the potential to induce the vulnerability of WM in the right hemisphere requires further investigation.

The findings of the current study have clinical significance. Studies have confirmed that alterations in WM integrity are reversible after certain types of physical or pharmacological treatment^36^,^37^. Administering long-chain polyunsaturated fatty acids to infants born to a methadone-maintained mother, which is essential for the formation of myelin, demonstrates a beneficial effect on their problem-solving skills and recognition-memory^38^. Therefore, the enhancement of myelin integrity may help to ameliorate the cognitive decline or to decrease the impulsiveness-related relapse in MMT patients.

The current study has several limitations. First, a major limitation is the lack of comparison groups in present study. Second, the version of FMRIB Software Library (FSL) used in this study is quite old. Third, possible gender-related differences of methadone effects on WM were not examined, since there were only three female MMT patients involved. Previous research has reported the existence of gender-related variations in opioid responses^39^. A sexual dimorphism in the oligodendrocytes and the WM of rodents has also been reported^40^. Therefore, the potential of a gender-related difference in methadone-related WM impairment requires further investigation. Finally, due to the lack of a detailed cognitive test or relevant functional imaging data in the present study, the conclusion that methadone-related reduction in WM integrity may underlie the neural substrates of the relevant cognitive deficits and the functional alterations of MMT patients remains a presumption. Further research to understand the correlation of WM integrity with cognitive deficits and functional alterations is strongly recommended.

In conclusion, convincing evidence was obtained about the methadone-induced impairment of WM structure using DTI. This impairment can be regarded as reflecting the neurotoxic effects of chronic methadone use, which may contribute to the origins of impaired brain function and cognitive function in MMT patients. The pattern of methadone-related WM impairment may shed light on the development of new approaches to protect WM integrity and to enhance therapeutic effectiveness.

## Methods

The present study was approved by the Institutional Board of the Fourth Military Medical University, Xi’an, China, including all the relevant details. As required by the Institutional Review Board of the Tangdu Hospital, all subjects were informed of the experimental details and aims of the study, and written consent for their involvement was obtained from each subject. The experiment was carried out in accordance with the approved guidelines.

### Subject selection

Fifty right-handed former chronic heroin addicts under stable MMT were recruited from the outpatients of Baqiao Methadone Maintenance Treatment Center, Xi’an, China. All of them voluntarily came to the MMT center to seek medical help. The primary inclusion criteria for heroin addicts were: (1) Diagnosed with heroin addiction as defined by the DSM-IV (American Psychiatric Association) diagnostic criteria; (2) Under MMT for at least three months with a stable dose; (3) Being right-handed (as judged by the Edinburgh handedness inventory); and (4) The primary route of heroin administration was snorting. Individuals were excluded if they had: (1) A drug abuse history other than heroin addiction; (2) Any history of head trauma and/or neurological disease or neurological disease signs; (3) Intelligence and language communication issues; (4) Daily alcohol consumption; (5) A current medical illness; (6) Claustrophobia or any contraindication for MRI examination.

### Neuropsychology status assessment

The co-morbidity of addiction and psychiatric disorders is commonly observed, such as depression, which has been observed in 42–54% MMT patients^41^. Therefore, the Beck Depression Inventory II (BDI) and the Hamilton Anxiety Scale (HAMA) were used, respectively, to evaluate co-morbid depression and anxiety. In addition, the Barratt Impulsiveness Scale (BIS-11), which is composed of three factors, was used: Attentional impulsiveness, motor impulsiveness and NPI, were used to explore the potential correlation between WM integrity and impulsiveness.

### Follow-up design and setting

A longitudinal self-control design was used in the present study. All participants underwent two MRI scans, which were conducted one year apart. The first scan was arranged at the initiation of the study in October 2012, and the second one was at the end of a follow-up in October 2013. Information regarding MMT consumption during the one year period between the two scans was recorded. Any occasional heroin consumption was determined through a regular interview and monthly urine test.

### Image acquisition

All images were acquired on a 3.0 T MR scanner (GE Signa Excite HD) in the Imaging Center of Tangdu Hospital, with a gradient strength of 40 mT/m and a slew rate of 150 T/m/sec. Routine T_2_-weighted imaging was acquired and carefully examined by an experienced radiologist to ensure the absence of gross structural abnormalities. A spin-echo echo planar imaging sequence was used in DTI image acquisition. During each scanning session, 34 axial slices covering the whole brain were acquired with the following parameters: repetition time = 7,600 ms, echo time = 61.5 ms, matrix = 128 × 128, field of view = 240 × 240 mm^2^, number of excitations = 2 and slice thickness = 4 mm, with no gaps. The diffusion images’ gradient encoding schemes included 25 non-colinear orientations with a b-value = 1000 s/mm^2^ and a non-diffusion weighted image with a b-value = 0 s/mm^2^. The total scanning time for each subject was approximately 10 minutes. Ear plugs and foam padding were used to reduce noise and minimize head motion.

### DTI data processing

DTI data were pre-processed using the Diffusion Toolbox (FDT) from the FMRIB Software Library (FSL version 4.1.8, http://www.fmrib.ox.ac.uk/fsl/)^42^. The DTI data of each individual was first corrected for eddy current distortion and head motion by FDT. The Brain Extraction Tool (BET) was used to remove background noise and the non-brain tissue components^43^. Afterwards, the parameter maps, including fractional anisotropy (FA) and three eigenvalues (λ_1,_ λ_2_ and λ_3_) were obtained using DTIFIT from FSL. Then, the axial diffusivity (AD = λ_1_) and radial diffusivity (RD = [λ_2_ + λ_3_]/2)^44^ were calculated with an in-house program.

Whole brain voxel-wise statistical analyses of FA images were performed by standard Tract-Based Spatial Statistics (TBSS) implemented in FSL^42^,^45^. First, the FA map of each subject was realigned to the standard FMRIB58_FA template in Montreal Neurological Institute (MNI) space and re-sampled to 1 × 1 × 1 mm^3^ 46. Afterwards, registered FA images of all the subjects were averaged to generate a cross-subject mean FA image and were skeletonized to create a mean FA skeleton by thinning the mean FA image to represent the center of all tracts common to the whole group. The threshold of the mean FA skeleton was set to 0.2 in order to exclude peripheral tracts. The aligned FA data of each participant was then projected onto the group mean skeleton to create a skeletonized FA map by filling the skeleton with the FA values from the nearest relevant tract center. In addition, the RD and AD maps were projected onto the skeleton using the same non-linear transformation matrix.

### Statistical analysis

Voxel-wise statistical analyses were performed to investigate the FA, AD and RD differences between the two scans, which were derived from the Randomise tool in FSL (http://www.fmrib.ox.ac.uk/fsl/randomise/index.html) to illustrate the nonparametric permutation inference of the data. The number of permutation tests was programmed as 5,000 by using Randomise (routine in FSL)^47^. The single-group paired difference (paired t-test) was provided by the GLM in FSL and was used to generate the design matrix and contrast files for the comparison of the two scans. Threshold-free cluster enhancement (TFCE) was used to define the clusters demonstrating significant differences between the two scans. The results were then corrected for multiple comparisons with a family-wise error (FWE) and the criterion set as *P* < 0.05^48^. The skeletal regions showing a significant intergroup difference were defined by the Johns Hopkins University (JHU)-ICBM-DTI-81 WM labels atlas and the JHU white matter tractography atlas in MNI space by using the atlasquery tool in FSL.

A paired t-test was performed to test the difference in the neuropsychological scores between Scan_1_ and Scan_2_. Pearson correlations were performed to explore the relationship between the proportion of change in FA, AD and RD in overlapping regions and the accumulated dose of methadone use and the occasional use of heroin within one year. The associations between the changes of the DTI indices and the BDI, HAMA and BIS scores were also determined by Pearson correlations using the Statistical Package for the Social Sciences version 16.0 (SPSS Inc., Chicago, IL, USA). The criterion of statistical significance was set at *P* < 0.05.

## Additional Information

**How to cite this article**: Li, W. *et al*. Methadone-induced Damage to White Matter Integrity in Methadone Maintenance Patients: A Longitudinal Self-control DTI Study. *Sci. Rep.* **6**, 19662; doi: 10.1038/srep19662 (2016).

## Figures and Tables

**Table 1:** Demographic and clinical characteristics of the participants (Mean ± SD).

Characteristics	MMT patients (N = 33)
Age (years)	34.8 ± 7.7
Gender(M/F)	30/3
Educational level (years)	9.3 ± 2.4
Dosage of cigarette smoking (cigarettes/day)	21.2 ± 5.2
Duration of cigarette smoking (years)	16.5 ± 8.3
Accumulated dosage of former heroin abuse (g)	951.9 ± 1464.9
Duration of former heroin abuse (months)	76.3 ± 66.5
Accumulated dosage of former methadone use (l)	35.8 ± 29.7
Duration of former methadone use (months)	26.2 ± 16.6
Maintenance dosage of MMT (ml/day)	44.7 ± 16.2
Heroin use during the interval between the two scans	
Proportion of heroin relapse (relapse/non-relapse)	19/14
Average frequency of heroin relapse	3.3 ± 3.6
Average accumulated dosage of heroin use (g)	1.1 ± 1.3

**Table 2:** 

	S_1_ (N = 33)	S_2_ (N = 33)	t-value	*P*-value
BDI score	9.70 ± 8.63	8.93 ± 9.28	0.73	0.47
HAMA score	8.52 ± 11.05	10.94 ± 10.46	−2.36	0.02※
Total BIS score	62.76 ± 8.13	62.51 ± 7.83	0.14	0.89
Attentional impulsivity	13.42 ± 3.04	14.27 ± 2.81	−1.09	0.28
Motor impulsivity	19.90 ± 3.77	19.24 ± 3.84	0.87	0.39
Non-plan impulsivity	29.33 ± 4.80	28.91 ± 4.24	0.39	0.69

Neuropsychological scores of MMT patients when scanned (Mean ± SD). ^※^Significant different, *P* < 0.05; S_1_ = Scan_1_, S_2_ = Scan_2_.
